# Low Molecular Weight Chitosan-Insulin Complexes Solubilized in a Mixture of Self-Assembled Labrosol and Plurol Oleaque and Their Glucose Reduction Activity in Rats

**DOI:** 10.3390/md16010032

**Published:** 2018-01-16

**Authors:** Amani M. Elsayed, Aseel H. Khaled, Mayyas M. Al Remawi, Nidal A. Qinna, Hussam Abu Farsakh, Adnan A. Badwan

**Affiliations:** 1 Department of Pharmaceutics, College of Pharmacy, Taif University, Taif 26571, Saudi Arabia; amanimoselsayed2015@gmail.com; 2 The Jordanian Pharmaceutical Manufacturing Co., Naor 11710, Jordan; aseel.k@jpm.com.jo; 3 Faculty of Pharmacy and Medical Sciences, University of Petra, Amman 11196, Jordan; myyas_nj@yahoo.com (M.M.A.R.); nqinna@uop.edu.jo (N.A.Q.); 4 Department of Pathology, First Medical Laboratory, Amman, Jordan; f1Lab@yahoo.com

**Keywords:** chitosan, insulin, oral, solubilization

## Abstract

Oral insulin delivery that better mimics physiological pathways is a necessity as it ensures patient comfort and compliance. A system which is based on a vehicle of nano order where positively charged chitosan interacts with negatively charged insulin and forms a polyelectrolyte complex (PEC) solubilizate, which is then solubilized into an oily phase of oleic acid, labrasol, and plurol oleaque-protects insulin against enzymatic gastrointestinal reduction. The use of an anionic fatty acid in the oily phase, such as oleic acid, is thought to allow an interaction with cationic chitosan, hence reducing particle size. Formulations were assessed based on their hypoglycaemic capacities in diabetic rats as compared to conventional subcutaneous dosage forms. 50 IU/kg oral insulin strength could only induce blood glucose reduction equivalent to that of 5 IU/kg (1 International unit = 0.0347 mg of human insulin). Parameters that influence the pharmacological availability were evaluated. A preliminary investigation of the mechanism of absorption suggests the involvement of the lymphatic route.

## 1. Introduction

Indigenous glucose homeostasis is a tightly regulated process. Carbohydrate consumption induces the secretion of insulin into the bloodstream. Insulin is a key player in regulating blood glucose mainly via facilitating its conversion into energy. If blood glucose drops below a threshold, our bodies compensate either by further carbohydrate metabolism or by glycogen conversion. When insulin is not secreted in sufficient amounts for maintaining this balance, external substituents must be supplied [1]. Currently, insulin is commonly administered to the body subcutaneously as this provides higher biopotency and dose precision. However, the influence of subcutaneous insulin on resistance is still unknown. Injections, nonetheless, have limitations including: lipoatrophy, and local allergic reactions such as erythema and pruritus. Alternative methods of insulin administration are under extensive investigation [2,3]. Various strategies have been applied to develop bioactive insulin formulations which can be delivered through buccal, nasal, rectal routes, etc. [4,5,6]. The oral route remains a more potent yet challenging alternative as aggressive GIT conditions need to be overcome through chemical and physical modifications of either the insulin molecule itself [7,8,9] and/or its surrounding microenvironment [10,11,12]. Unfortunately, none of the attempted preparations improved oral insulin absorption adequately to be of significant clinical benefit. 

One of the most interesting polymer carriers that have been employed for oral delivery of insulin is chitosan; practically, a heterogeneous mixture of a range of molecular weight molecules capable of forming polyelectrolyte complexes with the oppositely charged moiety. The drawback of such complexes is that they easily dissociate in acidic media since both chitosan and insulin are soluble at a pH value as low as that of the stomach. Sadegi et al. observed an efflux of insulin, in 0.1 N HCl, from nanoparticles encompassing polyelectrolyte complexes of trimethyl chitosan. To resolve the issue, chitosan-insulin complexes were solubilized within an oily matrix of oleic acid and anionic surfactants forming transport vesicles [13,14,15,16]. Our intended formulation would relate to a universal particulate dispersion of nano order in which insulin is dissolved and enwrapped by a unique polymer membrane, namely, chitosan which enhances solubilized insulin stability and allows controlling particle size, surface properties, and release rate. It serves a dual action of protecting insulin from digestive reduction as well as promoting its absorbance across the epithelium, and potentiates sustained release features. This approach was successfully tested on human subjects using laboratory scale preparations [17].

In order to further explore such dosage form, we, herein, put forth an integrated nano-sized preparation that would be suitable for oral delivery and provide superior protection for insulin against gastrointestinal conditions both in vitro and in situ. We studied the effect of varying chitosan molecular weight and degree of deacetylation on system integrity, chemical and physical stability and in vitro gastric protection. A method that mimics gastrointestinal conditions was developed for the in vitro assessment of the protection that our formulation provides to the insulin solubilizate. In addition, we addressed the relevance of using a solubilization approach over high pressure homogenization. Preparations of two different doses of insulin, as described latterly, were administered orally to streptozotocin (STZ) diabetic rats, the corresponding pharmacokinetic parameters were calculated, and a preliminary absorption mechanism was sought. 

## 2. Results and Discussion

Not only is oral delivery of insulin a convenient method to patients, it more importantly simulates the pathway of indigenous insulin in the body. This avoids any imbalances in insulin levels between portal and peripheral veins that can otherwise be caused by subcutaneous administration [5]. Moreover, oral administration ensures the pass of insulin through the liver, which helps stabilize glucose homeostasis. The principal of glucose homoeostasis must, therefore, be taken into account in oral insulin formulations in order to maintain its fundamental function at its normal mode. 

The rationale behind our current formulation was to prepare an uncharged lipo-vesicle to deliver insulin through and protect it from unfavourable gastro-intestinal conditions. Such particulates must be of nano order to penetrate the biolipid membrane. In a previous study, we have provided evidence for the formation of the assumed lipo-vesicle [17]. In the current study, we verified the various parameters of formulation and production, and investigated the pharmacokinetics and mechanisms involved. 

### 2.1. Formulation and Characterization of Nanoparticles

The interaction between chitosan and oleic acid within nanoparticles has been described elsewhere [15,17]. This type of interaction was previously reported by Demarger and Domard, where chitosan was found to be adsorbed as a monolayer on the surface of lipid particles [18]. This is consistent with the work of Hargreaves and Deamer [19], as well as Cistol et al., Walde et al. and Fukuda et al. [20,21,22,23], who also demonstrated that fatty acid composites are able to form vesicles within a particular pH range. It can, therefore, be speculated that our chitosan-oleic acid complex most likely behaves similar to a surfactant system. 

A significant improvement in the oral absorption of insulin can be achieved by rendering the protein hormone more lipo-philic through dispersion of insulin-chitosan complexes in oleic acid. The particle size of chitosan-insulin PEC dispersed in oleic acid was in the range the range of 2040 ± 63 nm. Reduction of the particle size of chitosan-oleic acid microspheres to nanosize was achieved by high pressure homogenization or by addition of surfactants. On the one hand, when the high pressure homogenizer was used, particle size dropped to 200 ± 12 nm. Chemical integrity of the encapsulated insulin was reserved after homogenization according to RP-HPLC-UV analysis. On the other hand, the use of surfactant mixtures produced particulates of 108 ± 9 nm (Table 1). 

**Figure 1:**
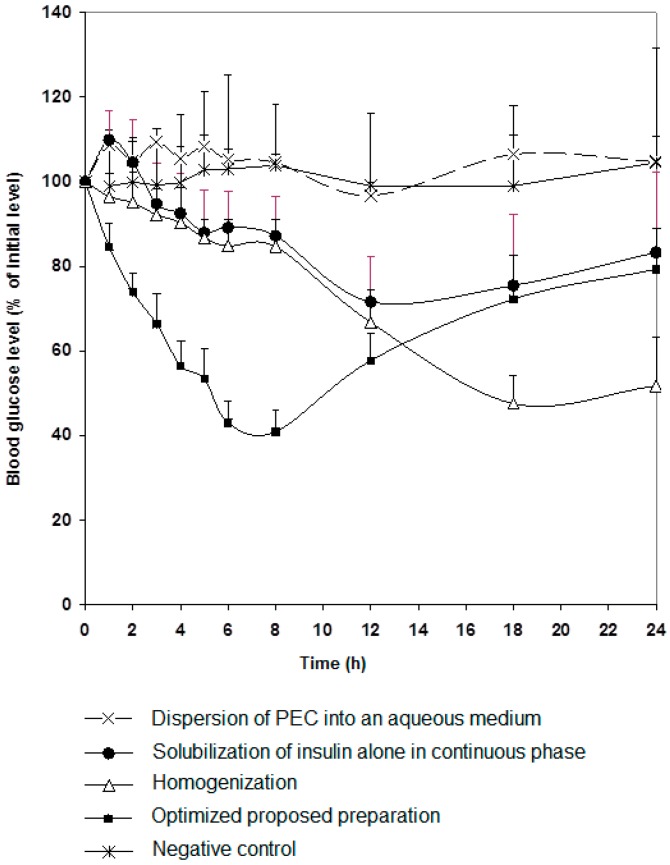
Changes in blood glucose level versus time profiles after single oral insulin administration (50 IU/kg) of different preparations (as in Table 1). Results are expressed as mean ± S.E.M (*n* = 10 per group).

It was clear, as anticipated, that both techniques successfully resulted in the production of particles though distinctly variant in terms of size. Both preparations were tested in vivo as shown in Figure 1. Rat blood glucose levels decreased remarkably post oral administration of the preparation containing surfactants, that could be significantly noticed at 3 h, compared to the control group (*p* < 0.05). More interestingly, the hypoglycemic effect was maintained without recovery for 24 h. In contrast, the preparation produced using high pressure homogenization induced a significant response (*p* < 0.001) only after 12 h of administration and the effect was sustained for 24 h. This indicates that chitosan-oleic acid nanoparticles were slowly absorbed compared to the preparation containing surfactants. Small particle size enables rapid absorption from the gastrointestinal tract, while larger particle size could be absorbed at a slower rate or at lower parts of the intestine [23]. The preparation that contained surfactants proved to be a more competent carrier for insulin. Further studies were carried out using this preparation. 

It is of importance to point out that the presence of chitosan significantly affects insulin protection as demonstrated in Figure 1, as reduction in glucose levels was not significant when a preparation without chitosan was given orally to rats. Effect of chitosan molecular weight and degree of deacetylation on degree of protection of insulin against gastrointestinal enzymes was tested in vitro to choose the most efficient form of the polymer. 

### 2.2. Measurement of the Protection Ability of Our Formula against Simulated Gastric and Intestinal Conditions In Vitro

The effect of chitosan molecular weight and degree of deacetylation on percent insulin protection of the formula in SGF was studied as shown in Table 2. Optimum insulin recovery (86.83 ± 3.98) after incubation in pepsin-infused SGF was achieved with 13 kDa chitosan of 99% DDA. This could be attributed to the presence of a higher number of NH_2_ groups available for protonation and subsequent interaction with both insulin and oleic acid. The interaction between chitosan and insulin was reported elsewhere [14]. Chitosan adsorbs as a monolayer on the surface of lipid particles [18]. Chitosan may, thereof, adsorb to the interphase surface due to its high affinity to oleic acid. The chitosan layer is, therefore, thought to confer protection upon and increase the stability of insulin against gastric enzymes.

The protective capacity of our nanoparticles (using Chitosan 13 kDa, 99% DDA) against SGF and SIF was further evaluated. Table 2 shows the amounts of insulin recovered from our formula after incubation in SGF, SIF and in SGF followed by SIF. It can be seen from the results that around 20% and 30% of insulin were digested in SGF and SIF, respectively, and that this was approximately equivalent to what was retained after incubating the formula in SGF followed by SIF as shown in Table 3.

### 2.3. Pharmacological Activity of Chitosan-Insulin Complex Given Subcutaneously to Diabetic Rats

**Figure 2:**
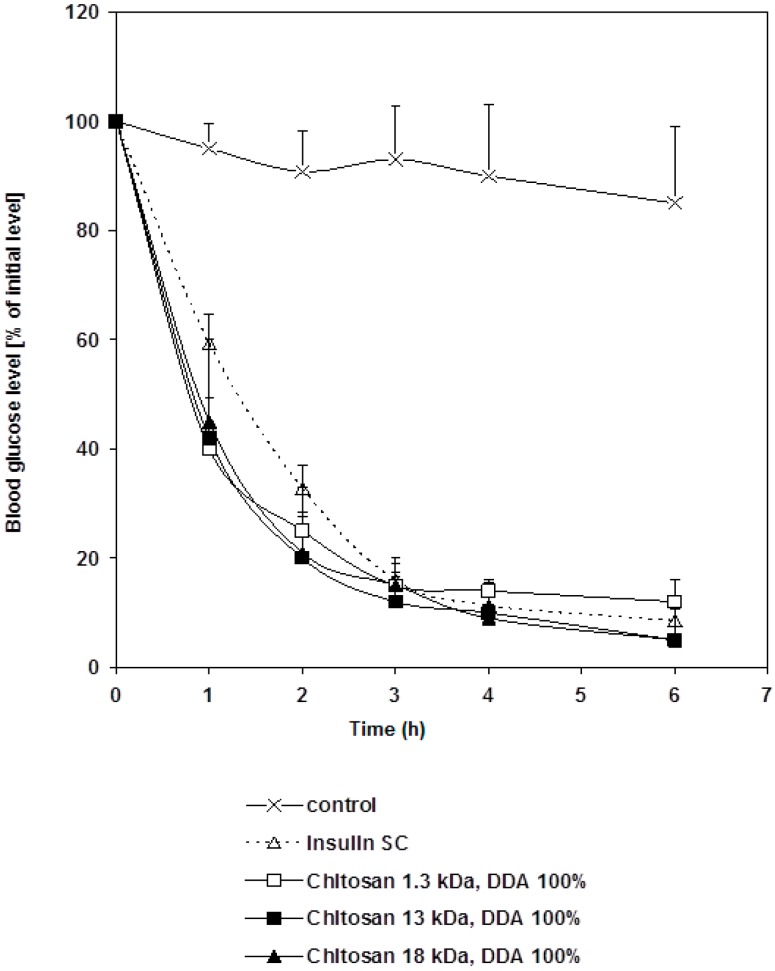
Changes in blood glucose level versus time profiles after a subcutaneous administration of free insulin and chitosan-insulin PEC given to STZ-diabetic rats at a dose of 1 IU/kg compared to a control group. Results are expressed as mean ± S.E.M (*n* = 10 per group).

Standard insulin solution and chitosan-insulin complex were injected subcutaneously into diabetic rats. There was no significant difference in glycemic effect (*p* > 0.05) between the two groups. However, there was a significant difference in glycemic effect (*p* < 0.01) between the control group and the treated groups as shown in Figure 2. This indicates that, the hypoglycemic effect of insulin was reserved and insulin activity did not change upon the electrostatic interaction with chitosan. Altering the molecular weight of chitosan did not change such behavior.

### 2.4. Bioavailability of Insulin-Loaded Nanoparticles

**Figure 3:**
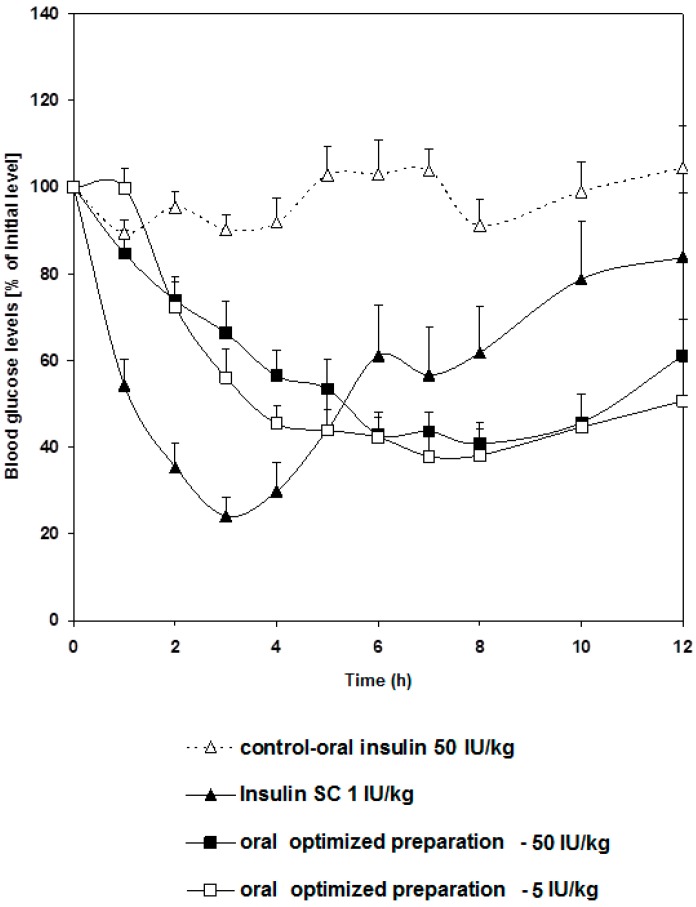
Changes in blood glucose level versus time profiles after a single oral administration of the optimized preparation given in two dose levels: 5 and 50 IU/Kg to STZ-diabetic rats compared to free insulin solution given orally in a dose of (50 IU/Kg) and used as a control group and another group given free insulin solution (1 IU/Kg) via subcutaneous injection. Results are expressed as mean ± S.E.M (*n* = 12 per group).

The pharmacological responses for 5 and 50 IU/kg of insulin delivered orally are shown in Figure 3. Apart from insulin amounts, each oral preparation contained the same components and had a similar particle size. The responses in reducing blood glucose level in diabetic rats were almost similar, which definitely does not reflect the dose difference. The lack of difference in the pharmacological response following administration of variable oral insulin doses was also reported by other groups [24,25]. For example, it was reported that rh-insulin microemulsion showed a non-linear dose-C_max_ relationship [26]. Such behavior may be attributed to the extent of oil solubilization by bile secretions. Other researchers explained the similarity in pharmacological response following administration of different oral insulin doses to the ability of the carrier polymers to bioadhere on mucus membrane where a restricted number of vesicles would penetrate the mucus layer in the intestine [27]. Generally, these reports summarize investigators’ points of view, which focus on the absorption process. None of these reports explore the physiological element in insulin hepatic metabolism. Ultimately, we took hepatic pass into consideration in our attempt to explain this phenomenon. 

**Figure 4:**
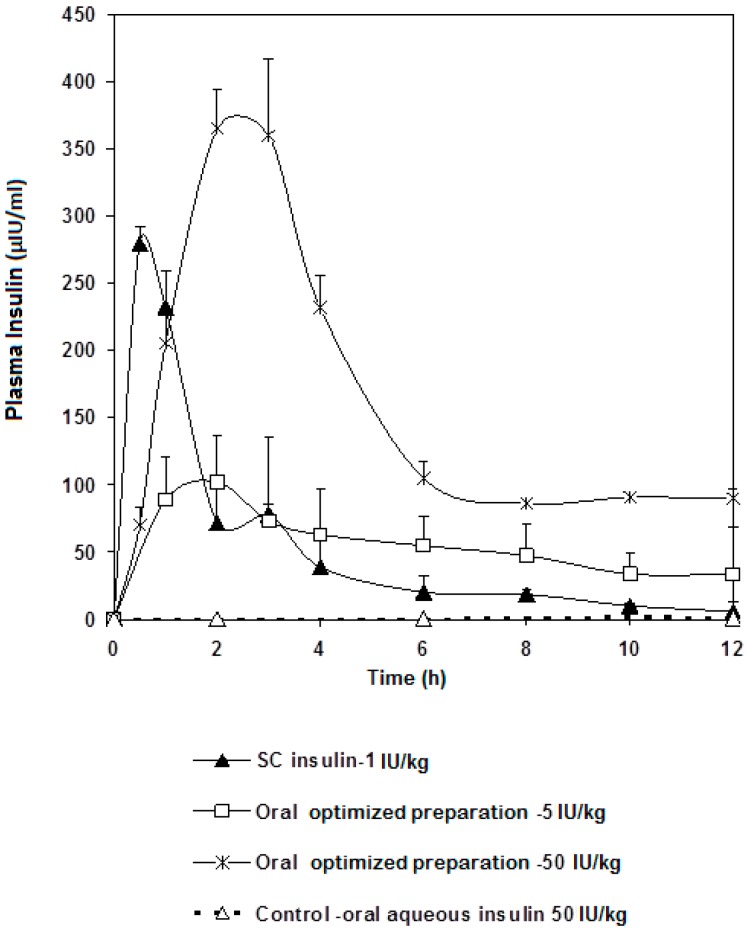
Insulin plasma levels profile after a single oral administration of the optimized preparation in doses of 5 and 50 IU/kg to fasted STZ diabetic rats compared to subcutaneous injection of free insulin (1 IU/Kg) and oral free insulin aqueous solution (50 IU/kg) as a control. Results are expressed as average of three independent experiments (*n* = 18).

Ingested insulin that reached systemic circulation was measured. The plasma insulin concentration time plot and pharmacokinetic parameters are presented in Figure 4 and Table 4, respectively. Our oral insulin dosage form was compared to that of subcutaneous route. Pharmacological activity was found to exhibit nonlinearity as a function of different oral insulin doses. Such results agree with perfusion studies on hepatic clearance of insulin [28], where insulin clearance was shown to depend on both circulating insulin concentration and liver handling capacity of insulin. Our work also provided evidence that insulin present in the plasma would not reduce the plasma glucose in a linear fashion. Such behavior may be due to the tightly-orchestrated glucose homeostasis mechanism, the minutiae of which are not well understood still. 

With that being said, the following could be noted from our plasma insulin profile: C_max_ of the 50 IU/kg dose was approximately 3.5 fold higher than that of the 5 IU/kg dose. Both oral doses showed T_max_ at 2 h, whereas the subcutaneous dose reached maximum concentration after 30 min. AUC were calculated and, as expected, that of the 50 IU/kg dose was higher, practically double, the 5 IU/kg dose (Table 4).

**Figure 5:**
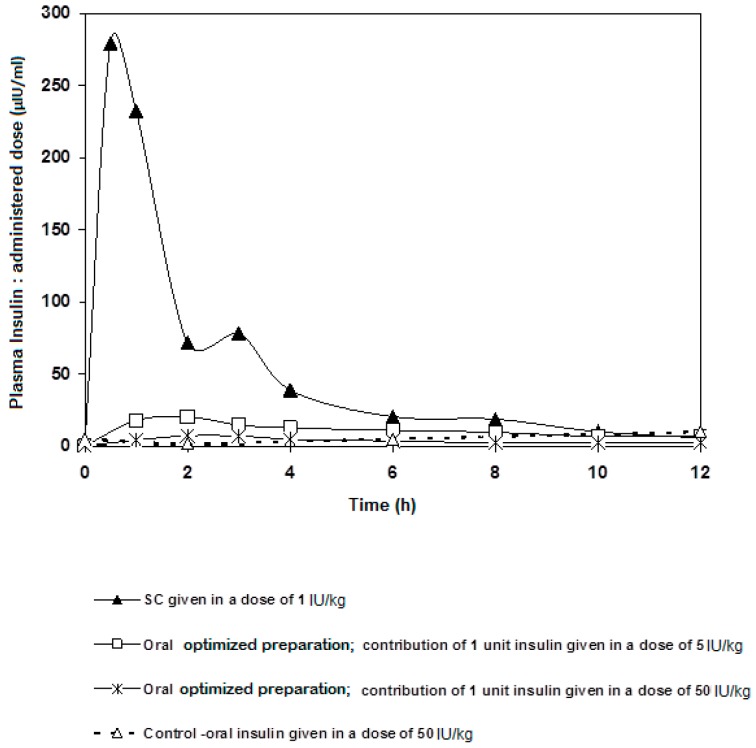
Contribution of a single oral unit of insulin (normalized) given in two different oral doses (5 and 50 IU/kg) compared to SC insulin given in a dose of 1 IU/kg.

To interpret the above results in a more fruitful manner, the plasma insulin concentrations were normalized against oral administered dose and plotted versus time, in order to observe the contribution of a single-Unit oral dose for each of the two doses; 5 and 50 IU/kg (Figure 5). Surprisingly, C_max_ and AUC that belong to a single unit of the 5 IU/kg dose were higher than their 50 IU/kg counterparts. Indeed, this can be explained by the liver’s ability to clear out insulin. Seemingly, insulin occurrence in the blood does not necessitate reduction in glucose concentration. In addition to the involvement of liver metabolism, it has been reported that diabetic rats showed more preserved glucose homeostasis with less hypoglycemic shocks seen in animals when injected with multiple doses of insulin: 1 IU/kg, 2 IU/kg, 4 IU/kg, and 6 IU/kg, compared to normoglycemic animals, despite the fact that insulin was found circulating in higher amounts in the blood of the STZ diabetic animals [29]. Results revealed that increasing the dose of injected insulin above 2 IU/kg did not elicit any further reductions in blood glucose levels (*p* > 0.05). This observation allows one to conclude that the design of an oral insulin dosage form requires taking into consideration the influence of hepatic metabolism, which highlights the need for more elaborate research. This study provides evidence that this controversy is not due to association of insulin to nanoparticles, but rather to the nature of insulin action as a hormone. It is well known that the number of insulin receptors on cell surfaces depends on plasma insulin concentration. When circulating insulin is high, more of the insulin receptors become occupied and the affinity of the receptors for insulin decreases. A low circulating level of insulin triggers the number of receptors to increase and enhances insulin binding [30].

Looking at the contribution of a single unit of insulin of each different dosage form, the AUC of the 5 IU/kg dose was around 29% of that of the subcutaneous single unit; an extremely valuable result to follow on for further studies. It was also remarkable that, as in Figure 5, insulin went down to its basic blood concentration after 6 h regardless the route of administration. Indeed, this piece of data can give a glimpse at the significance of rate of hepatic insulin clearance as far as peripheral glucose regulation is concerned. Usually, glucose ingestion causes changes in insulin clearance. Our results stress the need to incorporate hepatic delivery factors to criteria of formulating oral insulin. In conclusion, a preparation containing solubilized insulin-chitosan complex offers a competent carrier capable of delivering oral insulin at a range of doses while offering superior protection against the harsh conditions of the GI tract. It is noteworthy that blood glucose levels do not drop below a certain limit the higher the insulin doses given.

### 2.5. Overview of the Mechanism of Absorption

**Figure 6:**
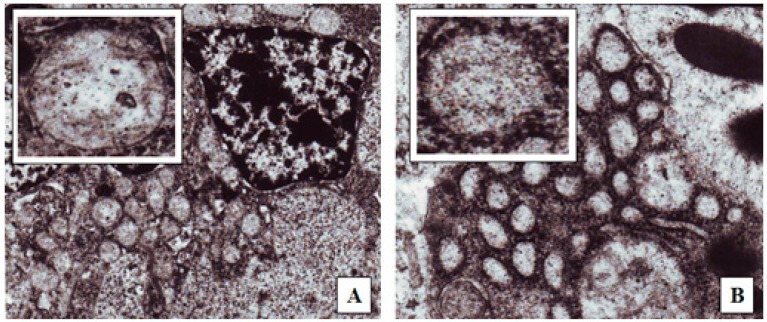
TEM (100,000×) images of rat duodenum after the administration of: (**A**) Distilled water; (**B**) Optimized insulin-loaded nanoparticle oily preparation. The upper left small pictures are enlargements of the endocytes seen in each picture.

**Figure 7:**
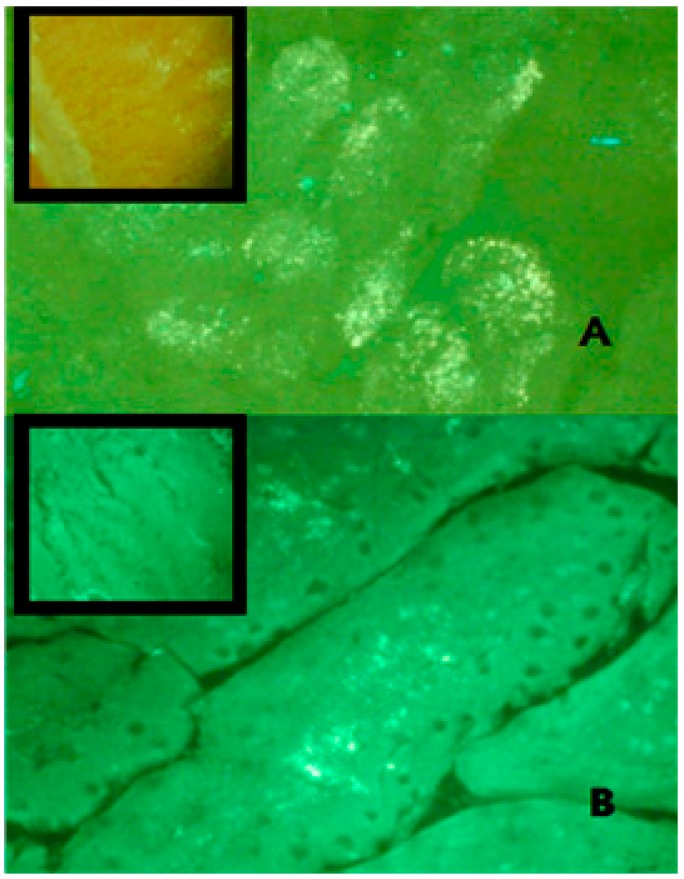
Frozen sections of rat duodenum administered with FITC-tagged insulin loaded nanoparticles investigated under a fluorescent microscope using a green filter (450–490 nm). Pictures were captured at 40× magnification at: (**A**) 1 h; (**B**) 2 h post administration. The fluorescence intensity was detected high in the duodenum of the rat 1 h post nanoparticle administration compared to the second hour sample. The oral administration of FITC-insulin aqueous preparation (upper left box) showed poor fluorescence intensity compared to the nanoparticle preparation.

A number of suggested mechanisms describe the uptake of macromolecules from the GI tract. Macromolecules can enter the intestine either paracellularly or transcellularly [31]. Transcellular transport of nanoparticles occurs by transcytosis, which begins with endocytosis that takes place at the cell apical membrane. Particles are transported through the cells and released at the basolateral pole [32]. Two types of intestinal cells are involved in nanoparticles transcytosis, namely, enterocytes and M cells, which are mainly located in the Peyer’s batches [33]. Endocytotic mechanisms include: pinocytosis, macropinocytosis or clathrin-mediated endocytosis [34]. Clathrin-coated vesicles internalize particles smaller than 150 nm, while in phagocytosis larger particles may be internalized [35]. Figure 6 shows TEM photos of the clathrin-coated vesicle for an epithelial cell of the gastrointestinal tract of nanoparticles taken via oral route compared with a control epithelial cell that does not engulf the nanoparticles. This may indicate that one of the proposed absorption mechanisms for nanoparticle transport is via a special type of endocytosis, particularly, clathrin-mediated endocytosis. This finding is consistent with a previous report in which chitosan nanoparticles were internalized by Caco-2 cell monolayer through clathrin-dependent pathways [36]. Further studies are needed, however. It is then important to determine the next route of uptake into systemic circulation; either portal or lymphati. Fluorescein-loaded nanoparticles given to normal rats were observed under the flouresent microscope and compared with control intestinal epithelium cells. Figure 7 shows strong fluorescein signals in the center of the intestinal villi. This may suggest that one transport mechanism which may control insulin transport to the systemic circulation could be through the lymphatic route [37]. However, under the same experimental condition and at the duodenal part of the gastrointestinal tract, control rats given oral fluorescein did not exhibit the same distribution of fluorescein signals. That being said, it can be claimed that the use of oleic acid as a vehicle would facilitate the lymphatic uptake particularly when the particle size is less than 150 nm. It was found that administration of osterdiol-3-cycloprntyl ether, instead, in aqueous or oily vehicle changes the mode of uptake [38]. As such, other routes of uptake cannot be ruled out and further exploration is needed. As a matter of fact, having several mechanisms of absorption may enhance bioavailability of the solubilized insulin, especially when the particle size is optimized. Presumably, hepatic clearance of insulin is the principal controlling factor in body protein utilization since passing through the liver after oral administration is inevitable.

## 3. Materials and Methods

### 3.1. Materials

Recombinant human insulin powder was purchased from Biocon, Bangalore, India. Low molecular weight chitosan was prepared by acidic depolymerization of high molecular weight chitosan (Xiamen Xing, Shanghai, China) via acid hydrolysis to produce low molecular weight chitosan (13 kDa and DDA 99%). The method of preparation was conducted according to previously published work [18]. Oleic acid was obtained from Merck, Darmstadt, Germany. Plurol oleique^®^ (polyglyceryl-6-dioleate) and Labrasol^®^ (PEG 8 caprylic/capric glycerides) were purchased from Gattefosse, Saint Priest, France. Strepotozotocin and Pepsin were obtained from Sigma-Aldrich, St. Louis, MO, USA.

### 3.2. Methods

#### 3.2.1. Chitosan Depolymerization and Deacetylation

Acid hydrolysis, according to Elsayed et al. [14], was applied to depolymerise chitosan of a fixed degree of deacetylation (99%) into different molecular weights: 1.3, 13, and 18 KDa. The method described by Alsouod et al. [39] was applied to obtain 13 KDa chitosan of different degrees of deacetylation: 55%, 80%, and 99%.

##### Preparation of the Oral Insulin Nanoparticle System

Aqueous phase: to prepare the PEC; (13 KDa, 99% DDA) chitosan was dissolved in water to give a 25 mg/mL solution of the pH 5.5, adjusted with 0.2 N NaOH. Recombinant human insulin was dissolved in 0.1 N HCl followed by the addition of 1 M TRIS (hydroxymethyl-aminomethane buffer pH 7.0) to give a 25 mg/mL solution. Equal volumes of the insulin and chitosan solutions were mixed gently for 15 min with frequent inversion. 

Continuous phase: to prepare the oily phase; 20 wt % non-ionic surfactants Labrasol (polyethylene glycol caprilic/capric glycerides) and Plurol Oleique (polyglecyryl-6-dioleate) at a weight ratio of (1:1) were suspended in 80 wt % of oleic acid. Insulin nanoparticles were obtained by dropping 500 µL of the PEC into 25 g of the oily phase and vortexing for 30 s at 25 °C. (6.6–7.1 IU) of insulin was delivered per mL of the formula (equivalent to 0.23–0.25 mg/mL). Control preparations involved modifying one or more of the parameters described above, and these include: (1) dispersion of the PEC into an aqueous medium; (2) Exclusion of chitosan from the PEC; (3) Addition of a final homogenization step (500 bars, 5 cycles) using a high pressure homogenizer (Emulsiflex-C5, Avestin Inc., Ottawa, ON, Canada) with cooling coil circulation for stability at a temperature not exceeding 35 °C; (4) negative control. 

#### 3.2.2. Characterization of Insulin Preparations

All preparations were characterized before administration to animals in terms of their insulin content and particle size [17].

#### 3.2.3. Insulin Content

Insulin was quantified by reversed phase high performance liquid chromatography (RP-HPLC) according to the USP 2007 method. The HPLC apparatus consisted of TSP 1000 pump, TSP 1000 UV-Vis detector, and a TSP AS 3000 autosampler (Spectra Systems, Providence, RI, USA). Chromatographic conditions: flow rate at 1 mL/min, wavelength (λ) at 214 nm, stationary phase of ACE 300 Ä (250 × 4.6 mm, 5 µm at 40 °C), mobile phase of 74:26 *v*/*v* 0.2 M Sulphate buffer pH 2.3: Acetonitrile, and injection volume of 100 μL [17].

#### 3.2.4. Particle Size Analysis

The particle size distribution of nanoparticles was determined using Zetasizer Nano-ZS (Malvern Instruments, Malvern, UK) at 25 °C.

#### 3.2.5. In Vitro Evaluation of Protection against Simulated Gastric Fluid (SGF) and Simulated Intestinal Fluid (SIF) of the Nano Formulation

The degree of protection was assessed in terms of percent insulin recovery against the gastrointestinal degradation. Enzymatic simulated gastric and intestinal fluids were prepared according to the US pharmacopeia 2007. In brief, 2 mL of the preparation was incubated with 5 mL of SGF for 15 min. followed by 5 mL of SIF for 30 min. After each incubation period, insulin was extracted from the formula using a solvent of (3:2) *v*/*v* methanol: 0.1 M HCl for HPLC analysis. 

#### 3.2.6. Procedure for Animal Preparation

Adult male Wistar rats with an average weight of 250 ± 50 g were accommodated under standard temperature, humidity and photoperiod light cycles. All rats were acclimatized for 10 days before experimenting day and received standard chow and tap water. All experiments were carried out following the European Community Council Directives 86/609/EEC and the study protocol was approved by the Ethical Committee of the Higher Research Council at the Faculty of Pharmacy, Petra University (Amman, Jordan).

Rats were made diabetic by administration of two intraperitoneal injections containing streptozotoscin (80 mg/kg) dissolved in 0.1 M citrate buffer of pH 4.5. Rats were checked for glucose blood levels using gluco-meter One Touch step™ (Life Scan, Inc., Milpitas, CA, USA). Blood was withdrawn from the tail vein. In this study, rats with fasting glucose level higher than 200 mg/dL were considered to be diabetic and were allowed for further testing.

#### 3.2.7. Comparison of Glucose Reduction by Insulin and Chitosan-Insulin Complex Injected Subcutaneously

Fasted STZ-diabetic rats were divided into 3 groups, each consisting of 10 rats; first group was injected with 1 IU/kg of insulin, second group was injected with chitosan-insulin with complexes equivalent of 1 IU/kg of insulin, whereas, third group was used as a negative control group.

The measured glucose levels at different time intervals were expressed as percentages of the initial glucose level, i.e., before injection.

#### 3.2.8. Comparison of Different Oral Preparations

Fasted STZ-Diabetic rats were used. The experiment consisted of 4 groups each containing 10 rats. Each preparation was administered intragastrically by gavages (50 IU/kg). Blood glucose was measured every hour up to 24 h. Rats were prevented from food and allowed to have tap water in the first 12 h of the experiment. Free access to food and water was only allowed after the passage of the first 12 h. 

#### 3.2.9. Comparison of the Effect of 5 and 50 IU/kg Doses Administered Orally

The reduction of glucose level was measured after oral administration to STZ-diabetic rats. The pharmacological effects of two doses, 5 IU/kg and 50 IU/kg, were compared. Animals were divided into four groups each consisting of 12 diabetic rats. Group 1 was given oral insulin aqueous solution (50 IU/kg) as a control. Groups 2 and 3 were given our oral preparations containing 5 and 50 IU/kg, respectively. Group 4 was given 1 IU/kg of insulin solution subcutaneously.

#### 3.2.10. Monitoring Insulin Levels in Rats Following Oral Administration of 5 and 50 IU/kg

The same procedure was repeated as mentioned in the previous section where the blood samples of the same size were withdrawn from rats and pooled at times of sampling. Each group consisted of 18 rats. Pooled blood was centrifuged (Allegro 21R centrifuge, Beckman Coulter, Brea, CA, USA) at 300 rpm for 10 min. The clear layer was aspirated and its insulin content was immunoassayed (Ins-ESIA BioSource Europe SA, Nivelles, Belgium). This kit was declared not to have any cross reactivity between the rat and human insulin. The results were obtained by measuring the optical density at 450 nm using Bio-Rad micro plate reader (Bio-Rad, Berkeley, CA, USA). The method followed was in accordance with the manufacturer’s manual^®^. 

#### 3.2.11. Pharmacokinetics and Relative Bioavailability Treatment

Linear trapezoidal method was used to calculate the total area under curve for insulin (AUC) from 0–12 h [40]. The relative bioavailability was calculated in comparison with subcutaneous injection. Maximum concentration (C_max_) of insulin plasma level and the time (T_max_), taken to reach C_max_, were obtained from insulin plasma concentration-time profile.

#### 3.2.12. Statistical Analysis

The arithmetic mean was obtained for all experiments. One way analysis of variance (ANOVA) was applied for Inter-groups comparison.

In these experiments when *p*-value was less than 0.05, the difference was considered significant.

#### 3.2.13. Mechanism of Intestinal Absorption

##### Fluorescent Microscopy

Insulin was labelled with fluorescein isothiocyanate (FITC) to enable the visualization of nanoparticles under a fluorescent microscope. Insulin labelling was done according to the instructions of the manufacture (Fluro-TrapTM, Fluorescein labelling kit, Innova Biosciences, Cambridge, UK). Labeled insulin was used to prepare chitosan-insulin complex that was dispersed into the oily vehicle to formulate the nanoparticles. The fluorescein-containing nanoparticle preparation was compared with an aqueous preparation diluted in phosphate buffer pH 6.8 instead of oleic acid. Both samples were given to fasted non-diabetic rats orally. Two rats were sacrificed under ether anesthesia after 1 and 2 h post-dosing. The duodenal part of small intestine was immediately removed and washed thoroughly with normal saline. Small sections from the duodenum were laid over a steel holder and covered with cryoprotectant. The holder was placed at −20 °C for 30 min. Samples were cut into 6μm sections using a cryostat (Slee cryostat-Germany), placed on coated slides and examined under fluorescent microscope (Nikon, Tokyo, Japan) using green filter (450–490 nm).

##### Transmission Electron Microscopy (TEM)

TEM imaging was performed for duodenal samples obtained from orally treated Wister rat with insulin-loaded nanoparticles in oily preparation compared to a control rat given only distilled water. After removing the samples from the gut (as described above), it was dipped immediately in 2.5% gluteraldehyde for maximum 24 h. Samples were fixed in 1% osmium tetraoxide for 1 h, washed and dehydrated using acetone serial dilution (from 30% to 100%). Samples were embedded in pure resin and dried. Resin blocks were cut under ultramicrotome and the sections were fixed over small grids and stained. The sections were examined under a transmission electron microscope Zeiss EM10CR (Carl Zeiss, Oberkochen, Germany).

## 4. Conclusions

A system prepared from nonionic surfactants; Labrasol and plurol oleaque, mixed at a 1:1 ratio and dispersed in oleic acid, is able to solublize insulin. Such a system only provides minimal, if any, protection to insulin in vivo. Incorporation of low molecular weight chitosan increases membrane rigidity thus giving rise to a less permeable solubilized system with the capacity to protect insulin from unfavorable conditions in the GI tract. Surprisingly, rats responded in the same manner to both 5 and 50 IU/kg of oral optimized preparation. Results of glucose level reduction are in concordance with our conclusions. The system acts as a delivery system for insulin and its absorption was found to be through the Clarithin-coated mechanism. We, herein, propose that nanocarriers constitute a preferred oral delivery system for drug peptides and proteins.

## Figures and Tables

**Table 1:** Particle size and content determination of different preparations used.

Control Modifications	Mean Diameter (nm) ± SD	Assay of Insulin * ± SD
Dispersion of PEC into an aqueous medium	8 ± 0.4	100 ± 5%
Solubilization of insulin alone in continuous phase	685 ± 16	100 ± 5%
Homogenization	2040 ± 63	101 ± 5%
Optimized proposed preparation	200 ± 12	100 ± 4%
Negative control	108 ± 9	99 ± 2%

SD: standard deviation. * Assay was determined based on the HPLC method of insulin which is can be found in the USP monograph, and was reported earlier in reference [18].

**Table 2:** Effect of varying chitosan molecular weight and degree of deacetylation on percent protection of the proposed preparation against simulated gastric fluid with pepsin (SGF).

Preparation	Percent Insulin Recovery ± SD
1.3 kDa MWt, 99% DDA	66.3 ± 0.44
1.3 kDa MWt, 80% DDA	77.99 ± 9.41
1.3 kDa MWt, 55% DDA	27.2 ± 1.6
13 kDa MWt, 99% DDA	86.83 ± 3.98
18 kDa MWt, 99% DDA	78.08 ± 2.2

MWt: Molecular weight of chitosan. DDA: Degree of deacetylation of chitosan.

**Table 3:** Percent protection of insulin in the optimized proposed formula.

Incubation Media	Percent Insulin Recovery ± SD
SGF	86.83 ± 3.98
SIF	73.9 ± 0.2
SGF followed by SIF	58 ± 0.75

SGF: US Pharmacopeia Simulated Gastric Fluid TS with pepsin; SIF: US Pharmacopeia Simulated Intestinal Fluid TS with pancreatin; SD: standard deviation.

**Table 4:** Pharmacokinetic parameters derived from the concentration time profiles of insulin given orally and subcutaneously.

Preparation	C_max_ (µg/mL)	T_max_ (h)	AUC_0–12_ (µIU.h/mL)	F %
S.C injection (1 IU/kg)	279.20	0.5	665.60	
Oral preparation (5 IU/kg)	102.22	2	968.15	29.09
Oral preparation (50 IU/kg)	356.20	2	2130.95	6.40

C_max_: maximum plasma concentration. T_max_: time at maximum plasma concentration. AUC: Area under the curve of time plasma concentration. F %: relative bioavailability based on AUC and compared with subcutaneous dose.
